# The effectiveness of digital health technologies for patients with diabetes mellitus: A systematic review

**DOI:** 10.3389/fcdhc.2022.936752

**Published:** 2022-10-24

**Authors:** Sebastian Stevens, Susan Gallagher, Tim Andrews, Liz Ashall-Payne, Lloyd Humphreys, Simon Leigh

**Affiliations:** ^1^ Research Department, Organisation for the Review of Care and Health Applications, Daresbury, United Kingdom; ^2^ Centre for Health Technology, University of Plymouth, Plymouth, United Kingdom; ^3^ Warwick Medical School (WMS), The University of Warwick, Coventry, United Kingdom

**Keywords:** diabetes mellitus, HbA1c, glycemic control, mobile apps, mhealth

## Abstract

**Introduction:**

Diabetes mellitus (DM) is a leading cause of morbidity and mortality worldwide. At the same time, digital health technologies (DHTs), which include mobile health apps (mHealth) have been rapidly gaining popularity in the self-management of chronic diseases, particularly following the COVID-19 pandemic. However, while a great variety of DM-specific mHealth apps exist on the market, the evidence supporting their clinical effectiveness is still limited.

**Methods:**

A systematic review was performed. A systematic search was conducted in a major electronic database to identify randomized controlled trials (RCTs) of mHealth interventions in DM published between June 2010 and June 2020. The studies were categorized by the type of DM and impact of DM-specific mHealth apps on the management of glycated haemoglobin (HbA1c) was analysed.

**Results:**

In total, 25 studies comprising 3,360 patients were included. The methodological quality of included trials was mixed. Overall, participants diagnosed with T1DM, T2DM and Prediabetes all demonstrated greater improvements in HbA1c as a result of using a DHT compared with those who experienced usual care. The analysis revealed an overall improvement in HbA1c compared with usual care, with a mean difference of –0.56% for T1DM, –0.90% for T2DM and –0.26% for Prediabetes.

**Conclusion:**

DM-specific mHealth apps may reduce HbA1c levels in patients with T1DM, T2DM and Prediabetes. The review highlights a need for further research on the wider clinical effectiveness of diabetes-specific mHealth specifically within T1DM and Prediabetes. These should include measures which go beyond HbA1c, capturing outcomes including short-term glycemic variability or hypoglycemic events.

## Introduction

Diabetes is a leading cause of morbidity and mortality worldwide (1–3). The past three decades have seen a dramatic increase in the number of adults living with diabetes, with the World Health Organisation (WHO) highlighting an increase in prevalence from 108 million in 1980 to 422 million in 2014 (4), and forecasts suggesting this could be as high as 700 million by as early as 2045 (5). Unfortunately, most people living with diabetes do not meet International Diabetes Federation (IDF) treatment targets of glycemic control, i.e., glycosylated haemoglobin (HbA1c) ≤7% for those with Type 2 diabetes (T2DM) (6) and <6.5% for those living with Type 1 diabetes (T1DM) (7).

When diabetes is poorly managed, it can result in systemic complications such as coronary heart disease, stroke, kidney failure, retinopathy, and foot ulcers (8), these complications can further progress to severe disabilities or even death. As such, the WHO estimate diabetes to be the ninth leading cause of death worldwide, with an estimated 1.5 million deaths as a direct result of diabetes (4). Additionally, diabetes workforce shortages (9, 10), limited public funding, and increasing secondary care backlogs all complicate this picture, often resulting in those with diabetes failing to obtain the right support at the right time (11).

With the prevalence of diabetes only expected to increase, and no clear and scalable solution to solve the supply side issues faced by healthcare systems, there remains an urgent unmet need for cost-effective and widely accessible strategies which can empower and motivate people with diabetes to adhere to best-practice diabetes self-care behaviours. Over the years, there has been a growing body of evidence to support the role of self-management in treating Type 2 diabetes mellitus (T2DM) (12, 13).

Digital health technologies (DHTs), which include mobile applications (apps) have been rapidly gaining popularity in the self-management of chronic diseases (14), particularly following the COVID-19 pandemic (15). Given their widespread availability, minimal barriers to access and often low cost, DHTs have been proposed as cost-effective tools to supplement clinician visits and deliver continuity of care to those who may struggle to access incumbent services. Specifically, the availability of these technologies for purposes including self-management of blood glucose (16), insulin dosing and adjustment (17) and dietary advice (18), has created further opportunities for self-management among patients with diabetes mellitus.

To date, there have been numerous systematic reviews of DHTs dedicated to the management of either Type 1 or Type 2 diabetes which report positive intervention effects (19–21). However, others have been less conclusive, with some reporting on the use of SMS messaging (22) and computer-based platforms (23), while many were also conducted several years ago (24, 25), limiting interpretation given the significant rate of technological change experienced in the past decade. It is therefore clear that differences in study design, intervention group, outcome measures and the specific functions and features of the technologies under consideration have led to widespread variation in the estimation of the impact of DHTs within diabetes.

The aim of this systematic review is to therefore summarise the available literature concerning the impact of using digital health technologies on laboratory confirmed HbA1c, for individuals diagnosed with T1DM, T2DM and Prediabetes.

## Methodology

### Design

In June 2020, we conducted a systematic search of randomised control trials published between 1 June 2010 and 1 June 2020 *via* the PubMed database. In addition, we manually searched reference lists and Google Scholar to identify further papers. The studies were screened and selected by two independent reviewers.

### Search strategy

After an initial pilot search, search terms listed in **Table 1** were constructed around i) ‘mHealth’, ii) ‘diabetes’ and iii) ‘clinical trials/RCTs’ including the medical terms derived from WHO’s Global Burden of Disease Report (26) and additional common terms associated with diabetes mellitus. Database searches were also supplemented with reference list searches to ensure sufficient coverage. A diabetes clinician also checked all search terms to ensure the accuracy of the search and sufficient coverage of the literature.

**Table 1:** Search terms.

“mHealth” OR “m-health” OR “app” OR “mobile application” OR “mobile-application” OR “mobile app” OR “mobile app” OR “smartphone” OR “cell phone” OR “cellphone” OR “cell-phone” OR “mobile-phone” OR “ehealth” OR “e-health” OR “e health” AND “T2DM” OR “mellitus” OR “T1DM” OR “diabetes” OR “diabetic” OR “diabetics” OR “diabetic’s” OR “pre-diabetes” OR “pre-diabetic” OR “pre-diabetics” OR “prediabetes” OR “prediabetic” OR “prediabetics” OR “pre diabetes” OR “pre diabetic” OR “pre diabetic*” OR “pre diabetic*” OR HbA1c OR “glycaemic control” OR “glycemic control” AND “Clinical Study” OR “Clinical Trial” OR “Clinical Trial” OR “Phase I Clinical Trial” OR “Phase II Clinical Trial” OR “Phase III Clinical Trial” OR “Phase IV Comparative Study” OR “Controlled Clinical Trial” OR “Randomized Controlled Trial”

### Study selection

Studies were selected through a two-stage process. Firstly, two reviewers (SS and SL) independently examined all identified titles and abstracts (facilitated through the online systematic review application, Rayyan) using pre-defined inclusion and exclusion criteria outlined below. The inter-reviewer agreement was sought through consensus. A process was in place to resolve any disagreements by a third reviewer; however, this process was not required. The full article was retrieved when a decision could not be made from the abstract alone. After the initial abstract screening, the full text of potentially relevant articles was retrieved and independently assessed for inclusion by two reviewers.

### Inclusion criteria

As outlined in **Table 2**, studies reporting on randomised control trials demonstrating the clinical effectiveness of diabetes-specific mHealth technologies in patients diagnosed with T1DM, T2DM or Prediabetes were included. Included studies were published between 2010 and 2020 to ensure only the most current information was included, given the rapid changes to the digital health landscape. Only studies published in the English language were included due to a lack of resources available to conduct reliable translation. Only studies that reported HbA1c as a patient outcome were included for this systematic review as HbA1c is the most widely used and most studied clinical outcome related to technological therapy for DM, including DHTs (27). Furthermore, we also excluded posters, commentary, protocols, theses, duplicates, and studies focused on the diagnosis of diabetes.

**Table 2:** Inclusion criteria.

Is the study published between 2010 and 2020? Yes (proceed)No (reject) Is the study available in the English language? Yes (proceed)No (reject) Is the study a Randomised Control Trial (RCT)? Yes (proceed)No (reject) Did the intervention involve the use of diabetes-specific digital health technology? Yes (proceed)No (reject) Were trial participants diagnosed with Type 1 Diabetes, Type 2 Diabetes or Prediabetes? Yes (proceed)No (reject) Does the article measure HbA1c as a patient outcome? Yes (proceed)No (reject)

### Data collection and analysis

We synthesised the studies according to outcomes because the clinical perspective focuses on improving individual outcomes through the intervention. Using a piloted data extraction form, one reviewer extracted the study characteristics of included articles.

To determine the change in HbA1c, we pooled appropriate studies with intervention groups (using mHealth interventions) and control groups (usual care) and calculated the difference in mean average. We included studies that reported changes in HbA1c as a percentage from baseline to the end of the study for intervention and control groups. The findings and author conclusions of articles reviewed were extracted and reported in a systematic format (see **Tables 3**, **4**).

**Table 3:** Characteristics of included studies.

Authors	Year	Diabetes Type	Study				Participants				Study Quality
			App (Intervention)	Control	Intervention (n)	Control (n)	Age (years, mean % and SD)	Gender (%)	Duration of Diabetes (years, mean % and SD)	Ethnic Groups (ethnic group, mean %)
Valentiner et al. (28)	2019	Type 2	InterWalk Smartphone App with additional support	InterWalk Smartphone App without additional support	18	19	Experiment; 66.7 ± 7.3 Control; 65.1 ± 6.4	Experiment; Male = 64.9 Control; Male = 70.3	N/A	N/A	Low
Heisler et al. (29)	2019	Type 2	Peer Coaching with eHealth educational tool (iDecide)	Peer Coaching alone	146	144	Experiment; 64.3 ± 9.7 Control; 62.1 ± 10.5	Experiment; Male = 96.6 Control; Male = 98.6	Experiment; 15.0 ± 10.2 Control; 15.3 ± 9.9	Experiment; African American = 63.4, White = 36.6 Control; African American = 61.8, White = 36.8 Other = 1.4%	Low
Wang et al. (30)	2019	Type 2	Continuous care for patients with type 2 diabetes using mobile health application	Traditional discharge nursing	60	60	Experiment; 45.1 ± 7.8 Control; 45.8 ± 8.3	Experiment; Male = 55.0 Control; Male = 51.6	N/A	N/A	Acceptable
Skrøvseth et al. (31)	2015	Type 1	Diabetes Diary (DD) Smartphone App	Usual Care	15	15	Experiment; 41.08 ± 13.5 Control; 38.33 +- 7.3	Experiment; Male = 33.33 Control; Male = 40.00	N/A	N/A	Low
Kooiman et al. (32)	2018	Type 2	Fitbit Zip and Online Lifestyle Programme	Usual care	40	32	Experiment; 56.8 ± 11.4 Control; 55.8 ± 11.4	N/A	Experiment; 15.5 ± 7.7 Control; 14.9 ± 5.3	N/A	Low
Yu et al. (33)	2019	Type 2	Smartphone App: Diabetes-Carer combined with Self-monitoring Blood Glucose	No Intervention	45	47	Experiment; 50.3 ± 10.4 Control; 56.2 ± 8.4	Experiment; Male = 66.0 Control; Male = 61.7	N/A	N/A	Low
Hansel et al. (34)	2017	Type 2	Patient E-Coaching Nutritional Support	Usual Care	60	60	Experiment; 57.6 ± 8.1 Control; 55.5 ± 10.3	Experiment; Male = 33.3 Control; Male = 33.3	N/A	N/A	Acceptable
Klee et al. (35)	2018	Type 1	Webdia Smartphone App	Usual Care	28	27	Experiment; 13.6 ± 2.4 Control; 13.7 ± 2.4	Experiment; Male = 75% Control; Male = 37%	Experiment; 7.5 ± 4 Control; 5.5 ± 3.25	N/A	Low
Agarwal et al. (36)	2019	Type 2	BlueStar Mobile App for Self-management	Usual Care	72	67	Experiment; 51.5 ± 10.6 Control; 52.1 ± 10.7	Experiment; Male = 55.0 Control; Male = 49.0	N/A	Experiment; Caucasian = 41.8. Non-Caucasian = 58.2 Control; Caucasian = 44.3, non-Caucasian = 53.0	Low
Sun et al. (37)	2019	Type 2	Smartphone App for Self-management for Older People	Usual Care	44	47	Experiment; 67.9 (66-71) Control; 68.04 (66-72)	Experiment; Male = 43.0 Control; Male = 38.0	Experiment; 11.19 ± 6.39 Control; 11.52 ± 7.73	N/A	Low
Kim et al. (38)	2019	Type 2	mDiabetes Smartphone App for Self-management	Paper Version	90	82	Experiment; 60.0 ± 8.4 Control; 56.7 ± 9.1	Experiment; Male = 55.60 Control; Male = 47.60	Experiment; 13.2 ± 8.0 Control; 12.5 ± 7.3	N/A	Low
Gunawardena et al. (39)	2019	Type 2	Smart Glucose Manager (SGM) Smartphone App	Usual Care	27	25	Experiment; 52 ± 12 Control; 53 ± 11	Experiment; Male = 63.0 Control; Male = 57.0	Experiment; 11 ± 6 Control; 11 ± 7	N/A	Acceptable
Kerfoot et al. (40)	2017	Type 2	Team Based Online Game for Veterans with T2DM	Offline civics game	277	229	Experiment; 59.62 ± 10.3 Control; 59.9 ± 9.4	Experiment; Male = 94.7 Control; Male = 93.0	N/A	N/A	High
Frias et al. (41)	2017	Type 2	Digital medicine offering (DMO) wearable sensor patches and a mobile device app	Usual Care	80	29	Experiment; 57.8 ± (SE) 1.1 Control; 61.6 ± (SE) 1.7	Experiment; Male = 44.0 Control; Male = 65.0	N/A	Experiment; Caucasian = 66.0, non-Caucasian = 44.0 Control; Caucasian = 66.0, non-Caucasian = 44.0	Low
Kleinman et al. (42)	2017	Type 2	Gather Health Smartphone App to Improve Medication Adherence and Frequency of Blood Glucose Self-Testing	Usual Care	44	46	Experiment; 48.8 ± 9.0 Control; 48.0 ± 9.5	Experiment; Male = 81.8 Control; Male = 58.7	Experiment; 10.0 (5-16) Control; 8.5 (4-14)	N/A	Acceptable
Quinn et al. (43)	2016	Type 2	Web Portal to manage T2DM	Usual Care	62	56	1) Age <55 Experiment; 47.3 ± 6.8 Age <55 Control; 47.4 ± 7.5 2) Age >55Experiment; 59.0 ± 2.9 Age >55 Control; 59.5 ± 2.8	1) Age <55 Experiment Male; 37.8 Age <55 Control Male; 62.1 2) Age >55Experiment Male; 68.0 Age >55 Control Male; 37.0	1) Age <55 Experiment; 6.8 ± 4.5 Age <55 Control; 8.9 ± 7.5 2) Age >55Experiment; 10.3 ± 5.8 Age >55 Control; 9.2 ± 6.0	1) Experiment Age <55; Black = 32.4, White = 54.1, Other = 13.5 Control Age <55; Black = 55.2, White = 37.9, Other = 6.9 2) Experiment Age >55; Black = 20.0, White = 76.0, Other = 4.0 Control Age >55; Black = 40.7, White = 55.6, Other = 3.7	Low
Crowley et al. (44)	2016	Type 2	Advanced Comprehensive Diabetes Care (ACDC) (telemonitoring)	Usual Care	23	23	Experiment; 60 ± 8.4 Control; 60 ± 9.2	Experiment; Male = 100 Control; Male = 92	Experiment; Median (IQR) 12 ± 13 Control; Median (IQR) 12 ± 9	Experiment; White = 52.0, African American = 48.0, Other = 0.0 Control; White = 32.0, African American = 60.0, Other = 8	Low
Kardas et al. (45)	2016	Type 2	COMODITY12 mHealth System	Usual Care	30	30	Experiment; 59.9 ± 5.31 Control; 59.0 ± 8.09	Experiment; Male = 57 Control; Male = 63	N/A	N/A	Low
Fukuoka et al. (46)	2015	Type 2	Mobile Phone–Based Diabetes Prevention Program (mDPP) App with Omron Pedometer	Omron Pedometer only	31	30	Experiment; 57.1 ± 9.1 (36–76) Control; 53.4 ± 8.7 (36–65)	Experiment; Male = 22.6 Control; Male = 23.3	N/A	Experiment: Asian = 33.4, Hispanic/Latino = 13.3, White =43.3, More than 1 race = 10.0 Control; Asian = 12.9, African American = 9.7, Hispanic/Latino = 9.7, White = 61.3, More than 1 race = 6.4	Acceptable
Block et al. (47)	2015	Prediabetes	Alive-PD Smartphone App and Email Support	Usual Care	163	176	Experiment; 55.0 ± 8.8 Control; 54.9 ± 9.1	Experiment; Male = 68.1 Control; Male = 69.3	N/A	Experiment; White = 66.9, Hispanic = 4.3, Asian = 25.2, Other = 3.7 Control; White = 68.2, Hispanic = 8.0, Asian = 16.5, Other = 7.4	High
Wayne et al. (48)	2015	Type 2	Connected Wellness Platform from NexJ Systems, Inc.	Usual Care	48	49	Experiment; 53.1 ± 10.9 Control; 53.3 ± 11.9	Experiment; Male = 35 Control; Male = 20	N/A	N/A	Acceptable
Shahid et al. (49)	2015	Type 2	Smartphone Intervention (App not specified)	Usual Care	220	220	Experiment; 48.95 ± 8.83 Control; 49.21 ± 7.92	Experiment; Male = 61.4 Control; Male = 61.4	N/A	N/A	Low
Greenwood et al. (50)	2015	Type 2	Care Innovations Health Suite online portal (Intel-GE Care Innovations, Roseville, CA, USA)	Usual Care	45	45	Experiment; 53.9 ± 10.4 Control; 57.5 ± 10.6	Experiment; Male = 75 Control; Male = 79	Experiment; 8.3 ± 5.5 Control; 8.1 ± 5.3	Experiment; White = 33, Hispanic = 7, Black/African American = 1, American Indian = 2, Asian/Pacific Islander = 3, Other = 2 Control; White = 31, Hispanic = 9, Black/African American = 2, American Indian = 1. Asian/Pacific Islander = 5. Other = 1	Acceptable
Drion et al. (51)	2015	Type 1	Diabetes Under Control (DBEES)	Usual Care	31	32	Experiment; 33 ± 23 Control; 35 ± 18	Experiment; Male = 65 Control; Male = 61	Experiment; 18 ± 17 Control; 15 ± 14	N/A	Low
Kirwan et al. (52)	2013	Type 1	Glucose Buddy Smartphone App	Usual Care	36	36	Experiment; 35.97 ± 10.67 Control; 34.42 ± 10.26	Experiment; Male = 52 Control; Male = 25	Experiment; 19.69 ± 9.64 Control; 18.19 ± 9.77	N/A	Low

**Table 4:** Trial results according to HbA1c (%) values.

Authors	Year	Diabetes Type	HbA1c (%) Experiment			HbA1c (%) Control		
			Baseline	Post Intervention	Change from Baseline	Baseline	Post Intervention	Change from Baseline
Valentiner et al. (28)	2019	Type 2	6.60	6.80	0.20	6.90	6.80	-0.10
Heisler et al. (29)	2019	Type 2	9.06	8.52	-0.54	9.10	8.55	-0.55
Wang et al. (30)	2019	Type 2	8.62	7.12	-1.52	8.68	7.92	-0.76
Skrøvsethet al. (31)	2015	Type 1	8.33	7.89	-0.44	8.06	7.49	-0.57
Kooiman et al. (32)	2018	Type 2	8.50	8.22	−0.28	8.60	8.60	−0.0
Yu et al. (33)	2019	Type 2	8.30	7.00	-1.10	8.70	7.70	-1.10
Hansel et al. (34)	2017	Type 2	7.16	6.86	-0.30	7.27	7.48	0.21
Klee et al. (35)	2018	Type 1	8.10	7.77	-0.33	8.10	7.89	-0.21
Agarwal et al. (36)	2019	Type 2	8.89	8.22	-0.67	9.03	8.41	-0.62
Sun et al. (37)	2019	Type 2	7.84	6.84	-1.00	7.88	7.22	-0.66
Kim et al. (38)	2019	Type 2	7.70	7.30	-0.40	7.80	7.74	-0.06
Gunawardena et al. (39)	2019	Type 2	9.50	7.20	-2.3	9.40	8.30	-1.10
Kerfoot et al. (40)	2017	Type 2	11.7	10.6	-1.10	11.6	10.9	-0.70
Frias et al. (41)	2017	Type 2	8.66	8.47	-0.19	8.28	8.54	0.26
Kleinman et al. (42)	2017	Type 2	9.40	7.90	-1.50	9.10	8.20	-0.80
Quinn et al. (43)	2016	Type 2	9.85	8.00	-1.85	9.15	8.45	-0.70
Crowley et al. (44)	2016	Type 2	10.5	9.20	-1.30	10.5	10.2	-0.30
Kardas et al. (45)	2016	Type 2	6.78	6.75	-0.04	6.84	6.78	-0.06
Fukuoka et al. (46)	2015	Type 2	5.83	5.73	-0.10	5.70	5.66	-0.04
Block et al. (47)	2015	Prediabetes	5.60	5.34	-0.26	5.60	5.42	-0.18
Wayne et al. (48)	2015	Type 2	8.69	7.88	-0.82	8.89	8.13	-0.76
Shahid et al. (49)	2015	Type 2	10.09	8.63	-1.46	9.85	9.36	-0.48
Greenwood et al. (50)	2015	Type 2	8.46	7.35	-1.11	8.16	7.46	-0.70
Drion et al. (51)	2015	Type 1	7.70	7.90	0.20	7.80	7.90	0.10
Kirwan et al. (52)	2013	Type 1	9.08	7.80	-1.28	8.78	8.58	-0.20

### Risk of bias

Quality assessment was conducted using the controlled trials risk of bias checklist by SIGN (53). One author conducted a full quality appraisal of all included studies following a high level of interrater reliability (100%) achieved between two reviewers during a sub-sample (10%), quality appraisal analysis of included articles. The methodological quality of the included studies was not the main focus of this systematic review; therefore, content relevance took precedence over methodological rigour.

## Results

### Literature search results

**Figure 1:**
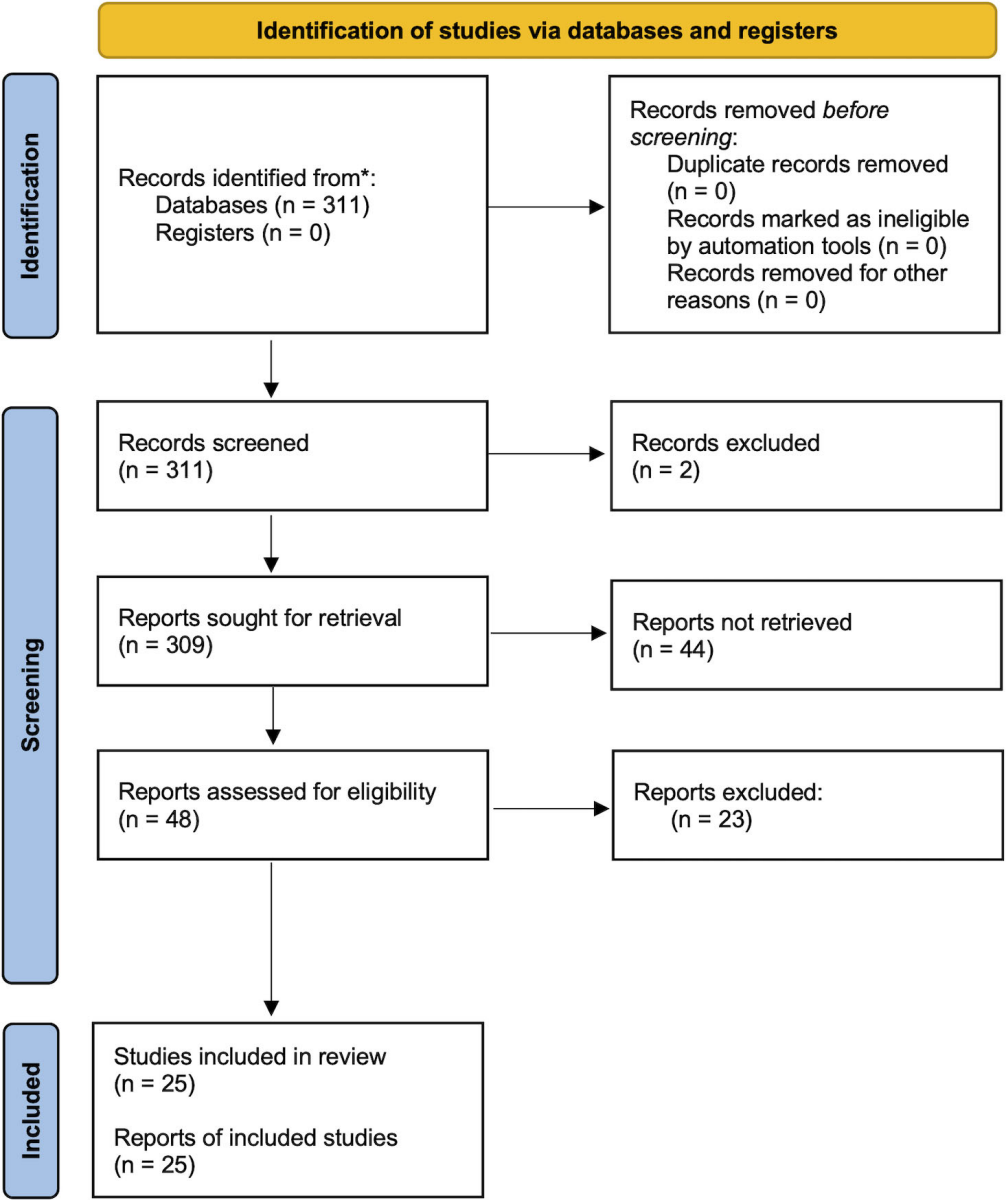
Overview of the screening process.

**Figure 1** illustrates the literature search and selection process. We identified a total of 311 studies from the PubMed database; no other studies were retrieved from citation searching. The full texts of 48 studies were screened, of which 23 were excluded due to the following reasons: i) they were not reporting the results of a mHealth intervention, iii) the study type was not an RCT (i.e., observational studies, systems reviews/meta-analyses, protocols, conference proceedings, theses), iv) the study did not report HbA1c as a patient outcome or v) other reasons. Twenty-five studies were ultimately included in our quantitative synthesis, with 4 focussed on T1DM, 20 studies on T2DM and 1 study on Prediabetes (see PRISMA flow diagram; **Figure 1**).

### Characteristics of included studies

#### Trial characteristics

**Table 3** summarises the main study characteristics of the 25 included trials. All studies included the outcome of HbA1c as either the primary or secondary outcome of the trial. As the review inclusion criteria required, all studies were randomised control trials, with different mHealth interventions evaluated in each clinical study. Additional patient outcomes reported in a number of these trials included (but were not limited to) blood markers (Fasting Blood Glucose/Fasting Plasma Glucose (FBG/FPG), 2-hour post-prandial blood glucose test (2-h PPG), 1,5-Anhydroglucitol test (1,5-AG)), triglycerides, cholesterol, blood pressure, insulin, aerobic capacity, body composition, hypoglycemic events, primary care visits, rates of rehospitalisation, and health-related quality of life.

A total of 3,360 participants were included in all 25 included studies, of whom 1735 received a mHealth intervention, and 1626 were included in a control group. The trial size varied from 30 to 440 participants. The mean age in the intervention group was 52.1 years and 52.0 years in the control group. Overall, females represented 39.4% of participants in the intervention group and 43.4% in the control group. Where reported, the average duration of diabetes in the intervention group was 12.49 years and 11.7 years in the control group.

#### Results of the RCT’s

**Table 4** shows the change in HbA1c both between the intervention and control groups across the 25 studies included in this review. Overall, a reduction in HbA1c was observed in 23 intervention groups and 21 control groups across the 25 included trials. Two trials observed an increase in HbA1c in the intervention group, with 4 trials observing an increase in HbA1c in the control group.

Overall, the intervention favoured the control group in 19 of the 25 trials, with 5 trials favouring the control group and 1 trial observing no difference between intervention and control groups. The combined pooled average reduction in HbA1c across the trial study periods was -0.80% across all the intervention groups and -0.45% within the control groups.

##### T1DM studies

Overall, 110 patients in the intervention groups and 110 patients in the control groups were investigated in the T1DM studies. Intervention favoured the control in 2 of the 4 studies (35, 52), with control favouring the other 2 studies (31, 51). Reduction in HbA1c levels was observed in 3 of 4 intervention groups (31, 35, 51), yielding a mean average reduction of -0.46% (**Table 5**).

**Table 5:** Trial results according to HbA1c (%) values pooled by mean average for T1DM, T2DM and Prediabetes.

	**HbA1c (%) Experiment**			**HbA1c (%) Control**		
**Diabetes Type**	**Baseline** **(Average)**	**Post Intervention** **(Average)**	**Change from Baseline** **(Average)**	**Baseline** **(Average)**	**Post Intervention** **(Average)**	**Change from Baseline** **(Average)**
Type 1 (n=4)	8.30	7.84	-0.46	8.19	7.97	-0.22
Type 2 (n=20)	8.61	7.73	-0.90	8.57	8.12	-0.47
Prediabetes (n=1)	5.60	5.34	-0.26	5.60	5.42	-0.18

##### T2DM studies

In total, 1,642 patients in the intervention groups and 1,340 patients in the control groups were investigated in the T2DM studies. Intervention favoured the control in 16 of 20 studies (30, 32, 34, 36–44, 46, 48–50) and no difference between intervention and control observed in one study (33). Reduction in HbA1c levels was observed in 19 of 20 intervention groups, with only one study observing an increase (28), yielding a mean average reduction of -0.90% (**Table 5**).

##### Prediabetes studies

Only one study investigated HbA1c levels for patients with Prediabetes (47). Within this study, 163 patients took part in the intervention groups and 176 patients participated in the control. Intervention favoured control in the study, with a reduction in HbA1c levels reported at -0.26% (**Table 5**).

#### Study quality

The methodological quality of included trials was mixed. Two studies were considered high quality (40, 47), 7 acceptable (30, 34, 39, 42, 46, 48, 50) and 16 low (28, 29, 31–33, 35–38, 41, 43–45, 49, 51, 52). Five of the 25 studies reported dropout rates above 20% before the study was complete (36, 38, 39, 48, 52) leading to possible under or overestimated clinical impact. Many of the studies highlighted as low quality were associated with issues with generalisability of the findings beyond the patient group under observation, challenges in generalising groups who are less motivated and technologically inclined and challenges to generalisability due to underpowering caused by sample size restrictions or dropout rates. Other common methodological weaknesses were associated with randomisation, concealment and blinding between subjects and assessors.

## Discussion

### Summary

The findings of this study, examining outcomes from a total of 3,360 participants across 25 RCTs, have demonstrated that mHealth interventions for those with diabetes mellitus can improve glycemic control. This was achieved by effectively reducing HbA1c values in patients with T1DM (mean difference: –0.46%), T2DM (mean difference: –0.90%) and Prediabetes (mean difference: –0.26%), with reductions in HbA1c levels observed 95% of the studies included. Studies were diverse with respect to the type of DM, study design, number of participants, and DHT functions and features. Often, different DHT features were combined, or the DHT was used in conjunction with web portals, feedback from HCPs, pedometers or other Bluetooth-enabled devices. Because of that, it was not possible to distinguish a relationship between specific DHT features and health outcomes.

### Interpretation considering other evidence

Overall, mHealth solutions were generally discussed to be feasible solutions to support the management of diabetes. Improvements in HbA1c in people with T1DM or T2DM observed in this review are consistent with the results of other reviews (27, 37, 54–58).

Alongside a reduction in HbA1c observed in the intervention group of the majority of included trials (as discussed above), a number of included trials also observed varying improvements in other patient outcomes including, but not limited to, fasting blood glucose, blood pressure, hypo and hyper glycemic events, cholesterol, body weight and self-care. Other reviews have analysed the impact on mHealth interventions of a number of these clinical outcomes (27, 37, 54, 57), however, unlike HbA1c, no consistent clinical impact has been identified.

A reduction in HbA1c was observed in 19 of the 20 intervention groups for T2DM, with only 1 study observing an increase (28), yielding an average reduction of -0.90% across all studies identified. This is similar to recent findings by Eberle et al. (27), who identified an average reduction in HbA1c of -1.1% for people with T2DM, however, others have observed smaller effects of -0.40% (54, 57) and 0.44% (58) when focussed on different patient populations and/or less recent publications.

Five of the 25 studies reported dropout rates above 20% before the study was complete (36, 38, 39, 48, 52), leading to possible under or overestimation of clinical impact. Observing rates of attrition above 20% in RCT’s for app-based interventions is consistent with a recent systematic review and meta-analysis that observed an average dropout rate of 40% in RCT’s of app-based interventions for chronic disease (59) compared with lower rates of attrition in trials of non-app-based interventions (60–62). Previous studies have demonstrated that up to 80% of all participants in mHealth interventions may engage in only minimal use of these interventions, defined as logging in to the service less than twice, and only a small fraction of users consistently use the intervention long term (63, 64). Low attrition rates characterised by perception of own health as poor (incentivizing the need to change) (65), those who wanted to be involved in their own health care (66) and those who are younger and have higher levels of health literacy (65, 67). Another association with low attrition was with those engaged in multiple interventions. Individuals engaged in internet or phone programs as well as apps were more likely to remain in research studies (68). These characteristics are an important consideration for clinicians in understanding those patients who are more likely to engage with DHT and also appreciating the potential for an additive value of DHT technology in complementing existing support, such as that of traditional healthcare.

Furthermore, one explanation for the modest improvements in HbA1c observed could be the experience level of those participating in the studies. Where reported, the average duration of diabetes in the intervention group was 12.49 years and 11.7 years in the control group.

This “learning effect” was captured in the finding that overall, a reduction in HbA1c was observed in 23 intervention groups and 21 control groups across the 25 included trials. Therefore, there is less likelihood of reducing HbA1c if people are already well acquainted with managing their condition. A study with a narrower focus on newly and recently diagnosed patients may yield different results.

Finally, we defined glycemic control as maintaining the blood glucose control within the normal or euglycemic blood glucose levels safely, i.e. no hypoglycemia or hyperglycemia. Although we observed overall improvements in HbA1c for people with T1DM or T2DM, HbA1c provides only an approximate measure of glucose control and does not address short-term glycemic variability or hypoglycemic events. This means that a raised or decline HbA1c may suggest an increase in hypoglycemic events which are associated with negative patient outcomes, including increased risks of non-fatal stroke, cardiovascular related death, and total mortality (69).

### Limitations

An important limitation of our review is that we excluded all studies that did not report the results of RCTs. Observational studies and non-randomized trials may provide important information useful for understanding the effectiveness of mHealth, as now suggested by regulators, including the National Institute for Health and Care Excellence (70).

Nevertheless, we opted for excluding these studies as non-randomized trial designs carry a greater risk of being affected by multiple known and unknown biases (71). Another limitation of the review process could have been that the review only reports on literature available at the time of the search and written in English. Although two experienced reviewers assessed the records, we cannot entirely rule out that we missed potentially relevant articles. Many of the primary studies were found to have low methodological quality and level of reporting, often impacted by randomisation, concealment, and blinding between subjects and assessors. However, it should be considered that randomisation, concealment, and blinding between subjects and assessors in digital health-based RCTs is challenging due to the nature of subjects receiving and potentially requiring support to use a digital health technology.

Furthermore, only one study included in this review focused on Prediabetes (47), so this result should be treated with caution. Additionally, articles focusing specifically on gestational diabetes were not included in this review. Finally, the effects of mHealth are likely to differ depending on the specific type of intervention, the specific disease, and the specific context. Due to the focus on diabetes-specific mHealth interventions for this review, it is not possible to draw firm conclusions on the effectiveness of mHealth interventions in general.

The mean age in the intervention group was 52.1 years and 52.0 years in the control group. Prior analyses have demonstrated how older age can impact digital proficiency, and therefore a study with a greater proportion of people living with DM, who can be of any age, may yield different results.

From the clinical perspective, we defined glycemic control as maintaining the blood glucose control within the normal or euglycemic blood glucose levels safely, i.e. no hypoglycemia or hyperglycemia. HbA1c provides only an approximate measure of glucose control and does not address short-term glycemic variability or hypoglycemic events. This means that a raised or decline HbA1c may suggest an increase in hypoglycemic events which are associated with an increased risk of non-fatal stroke, cardiovascular related death, and total mortality (69).

Finally, the methodological quality of included trials was mixed. Two studies were considered high quality, seven acceptable and 16 low. Studies reported as low in quality were often associated with issues with generalisability of the findings beyond the patient group under observation, challenges in generalising groups who are less motivated and technologically inclined and challenges to generalisability due to underpowering caused by sample size restrictions or dropout rates.

### Conclusion

Our review identified 25 studies reporting results of RCTs of mHealth interventions for patients with T1DM, T2DM and Prediabetes. Overall, this review demonstrates that diabetes-specific mHealth interventions may reduce HbA1c levels in patients with T1DM, T2DM and Prediabetes. The review highlights a need for further research on the wider clinical effectiveness of diabetes-specific mHealth specifically within T1DM and Prediabetes. These should include measures which go beyond HbA1c, capturing outcomes including short-term glycemic variability or hypoglycemic events.

## Data availability statement

The original contributions presented in the study are included in the article/Supplementary Material. Further inquiries can be directed to the corresponding author.

## Author contributions

SS, TA & SL conceived the review topic. SS and SL drafted the protocol, performed literature search and conducted data extraction and analysis. SG contributed clinical expertise. SS, SG, TA, LA-P, LH, and SL contributed to manuscript authorship and editing. All authors contributed to the article and approved the submitted version.

## Conflict of interest

The authors declare that the research was conducted in the absence of any commercial or financial relationships that could be construed as a potential conflict of interest.

## Publisher’s note

All claims expressed in this article are solely those of the authors and do not necessarily represent those of their affiliated organizations, or those of the publisher, the editors and the reviewers. Any product that may be evaluated in this article, or claim that may be made by its manufacturer, is not guaranteed or endorsed by the publisher.

## References

[1] Bertoni AG Krop JS Anderson GF Brancati FL . Diabetes-related morbidity and mortality in a national sample of U.S. elders. Diabetes Care (2002) 25(3):471–5. doi: 10.2337/diacare.25.3.471 11874932

[2] Khan MAB Hashim MJ King JK Govender RD Mustafa H Al Kaabi J . Epidemiology of type 2 diabetes - global burden of disease and forecasted trends. J. Epidemiol. Glob Health (2020) 10(1):107–11. 10.2991/jegh.k.191028.001 PMC7310804 32175717

[3] Lin X Xu Y Pan X Xu J Ding Y Sun X . Global, regional, and national burden and trend of diabetes in 195 countries and territories: an analysis from 1990 to 2025. Sci. Rep. (2020) 10(1):14790. doi: 10.1038/s41598-020-71908-9 32901098 PMC7478957

[4] World Health Organisation . Diabetes key facts (2021). Available at: https://www.who.int/news-room/fact-sheets/detail/diabetes.

[5] Saeedi P Petersohn I Salpea P Malanda B Karuranga S Unwin N . Global and regional diabetes prevalence estimates for 2019 and projections for 2030 and 2045: Results from the international diabetes federation diabetes atlas, 9th edition. Diabetes Res. Clin. Pract. (2019) 157:107843. doi: 10.1016/j.diabres.2019.107843 31518657

[6] Bajaj S . RSSDI clinical practice recommendations for the management of type 2 diabetes mellitus 2017. Int. J. Diabetes Dev. Ctries. (2018) 38:1–115. 29527102 10.1007/s13410-018-0604-7 PMC5838201

[7] NICE . Tighter blood glucose targets for people with type 1 diabetes (2019). Available at: https://www.nice.org.uk/news/article/stricter-blood-glucose-targets-for-people-with-diabetes#:~:text=To%20tackle%20this%2C%20updated%20guidelines,mol%20(6.5%25)%20or%20lower.

[8] Harding JL Pavkov ME Magliano DJ Shaw JE Gregg EW . Global trends in diabetes complications: a review of current evidence. Diabetologia (2019) 62(1):3–16. doi: 10.1007/s00125-018-4711-2 30171279

[9] Vigersky RA Fish L Hogan P Stewart A Kutler S Ladenson PW . The clinical endocrinology workforce: current status and future projections of supply and demand. J. Clin. Endocrinol. Metab. (2014) 99(9):3112–21. doi: 10.1210/jc.2014-2257 24940655

[10] Gallegos A . Medical experts say physician shortage goes beyond primary care. Washington DC, USA: AAMC Reporter (2014).

[11] GOV UK . Written evidence submitted by diabetes UK (CBP0042) . Available at: https://committees.parliament.uk/writtenevidence/38601/pdf/.

[12] Mikhael EM Hassali MA Hussain SA . Effectiveness of diabetes self-management educational programs for type 2 diabetes mellitus patients in middle East countries: A systematic review. Diabetes Metab. Syndr. Obes. (2020) 13:117–38. 10.2147/DMSO.S232958 PMC6968799 32021358

[13] Lee AA Piette JD Heisler M Janevic MR Rosland AM . Diabetes self-management and glycemic control: The role of autonomy support from informal health supporters. Health Psychol. (2019) 38(2):122–32. doi: 10.1037/hea0000710 PMC6442463 30652911

[14] Debon R Coleone JD Bellei EA De Marchi ACB . Mobile health applications for chronic diseases: A systematic review of features for lifestyle improvement. Diabetes Metab. Syndr. (2019) 13(4):2507–12. doi: 10.1016/j.dsx.2019.07.016 31405669

[15] Leigh S Daly R Stevens S Lapajne L Clayton C Andrews T . Web-based internet searches for digital health products in the united kingdom before and during the COVID-19 pandemic: a time-series analysis using app libraries from the organisation for the review of care and health applications (ORCHA). BMJ Open (2021) 11(10):e053891. doi: 10.1136/bmjopen-2021-053891 PMC8506045 34635531

[16] Yingling L Allen NA Litchman ML Colicchio V Gibson BS . An evaluation of digital health tools for diabetes self-management in Hispanic adults: Exploratory study. JMIR Diabetes. (2019) 4(3):e12936. doi: 10.2196/12936 31313657 PMC6664655

[17] Nimri R . Decision support systems for insulin treatment adjustment in people with type 1 diabetes. Pediatr. Endocrinol. Rev. (2020) 17(Suppl 1):170–82. 10.17458/per.vol17.2020.n.insulintreatmenttype1diabetes 32208561

[18] Azelton KR Crowley AP Vence N Underwood K Morris G Kelly J . Digital health coaching for type 2 diabetes: Randomized controlled trial of healthy at home. Front. Digit Health (2021) 3:764735. doi: 10.3389/fdgth.2021.764735 34901926 PMC8655126

[19] Nkhoma DE Soko CJ Bowrin P Manga YB Greenfield D Househ M . Digital interventions self-management education for type 1 and 2 diabetes: A systematic review and meta-analysis. Comput. Methods Programs Biomed. (2021) 210:106370. doi: 10.1016/j.cmpb.2021.106370 34492544

[20] Kebede MM Zeeb H Peters M Heise TL Pischke CR . Effectiveness of digital interventions for improving glycemic control in persons with poorly controlled type 2 diabetes: A systematic review, meta-analysis, and meta-regression analysis. Diabetes Technol. Ther. (2018) 20(11):767–82. doi: 10.1089/dia.2018.0216 30257102

[21] Eberle C Stichling S . Telemetric interventions offer new opportunities for managing type 1 diabetes mellitus: Systematic meta-review. JMIR Diabetes. (2021) 6(1):e20270. doi: 10.2196/20270 33724201 PMC8080418

[22] Sahin C Courtney KL Naylor PJ E Rhodes R . Tailored mobile text messaging interventions targeting type 2 diabetes self-management: A systematic review and a meta-analysis. Digit Health (2019) 5. doi: 10.1177/2055207619845279 PMC6481002 31041110

[23] Coughlin SS Williams LB Hatzigeorgiou C . A systematic review of studies of web portals for patients with diabetes mellitus. mHealth (2017) 3:23. doi: 10.21037/mhealth.2017.05.05 28736732 PMC5505929

[24] Hou C Carter B Hewitt J Francisa T Mayor S . Do mobile phone applications improve glycemic control (HbA1c) in the self-management of diabetes? a systematic review, meta-analysis, and GRADE of 14 randomized trials. Diabetes Care (2016) 39(11):2089–95. doi: 10.2337/dc16-0346 27926892

[25] Bonoto BC de Araújo VE Godói IP de Lemos LL Godman B Bennie M . Efficacy of mobile apps to support the care of patients with diabetes mellitus: A systematic review and meta-analysis of randomized controlled trials. JMIR mHealth UHealth. (2017) 5(3):e4. doi: 10.2196/mhealth.6309 28249834 PMC5352856

[26] GBD . Diseases and injuries collaborators. global burden of 369 diseases and injuries in 204 countries and territories, 1990-2019: a systematic analysis for the global burden of disease study 2019. Lancet (2019) 396(10258):1204–22. 10.1016/S0140-6736(20)30925-9 PMC7567026 33069326

[27] Eberle C Löhnert M Stichling S . Effectiveness of disease-specific mHealth apps in patients with diabetes mellitus: Scoping review. JMIR mHealth UHealth. (2021) 9(2):e23477. doi: 10.2196/23477 33587045 PMC7920757

[28] Valentiner LS Thorsen IK Kongstad MB Brinkløv CF Larsen RT Karstoft K . Effect of ecological momentary assessment, goal-setting and personalized phone-calls on adherence to interval walking training using the InterWalk application among patients with type 2 diabetes: A pilot randomized controlled trial. PloS One (2019) 14(1):e0208181–e. doi: 10.1371/journal.pone.0208181 PMC6328102 30629601

[29] Heisler M Choi H Palmisano G Mase R Richardson C Fagerlin A . Comparison of community health worker-led diabetes medication decision-making support for low-income Latino and African American adults with diabetes using e-health tools versus print materials: A randomized, controlled trial. Ann. Internal Med. (2014) 161(10):S13–22. doi: 10.7326/M13-3012 PMC4391371 25402398

[30] Wang Y Li M Zhao X Pan X Lu M Lu J . Effects of continuous care for patients with type 2 diabetes using mobile health application: A randomised controlled trial. Int. J. Health Plann Manage. (2019) 34(3):1025–35. doi: 10.1002/hpm.2872 31368137

[31] Skrøvseth SO Årsand E Godtliebsen F Joakimsen RM . Data-driven personalized feedback to patients with type 1 diabetes: A randomized trial. Diabetes Technol. Ther. (2015) 17(7):482–9. doi: 10.1089/dia.2014.0276 PMC4504254 25751133

[32] Kooiman TJM de Groot M Hoogenberg K Krijnen WP van der Schans CP Kooy A . Self-tracking of physical activity in people with type 2 diabetes: A randomized controlled trial. Computers Informatics Nursing: CIN. (2018) 36(7):340–9. doi: 10.1097/CIN.0000000000000443 29742550

[33] Yu Y Yan Q Li H Li H Wang L Wang H . Effects of mobile phone application combined with or without self-monitoring of blood glucose on glycemic control in patients with diabetes: A randomized controlled trial. J. Diabetes Investig. (2019) 10(5):1365–71. doi: 10.1111/jdi.13031 PMC6717828 30815973

[34] Hansel B Giral P Gambotti L Lafourcade A Peres G Filipecki C . A fully automated web-based program improves lifestyle habits and HbA1c in patients with type 2 diabetes and abdominal obesity: Randomized trial of patient e-coaching nutritional support (The ANODE study). J. Med. Internet Res. (2017) 19(11):e360–e. doi: 10.2196/jmir.7947 PMC5700402 29117929

[35] Klee P Bussien C Castellsague M Combescure C Dirlewanger M Girardin C . An intervention by a patient-designed do-It-Yourself mobile device app reduces HbA1c in children and adolescents with type 1 diabetes: A randomized double-crossover study. Diabetes Technol. Ther. (2018) 20(12):797–805. doi: 10.1089/dia.2018.0255 30403495

[36] Agarwal P Mukerji G Desveaux L Ivers NM Bhattacharyya O Hensel JM . Mobile app for improved self-management of type 2 diabetes: Multicenter pragmatic randomized controlled trial. JMIR mHealth uHealth. (2019) 7(1):e10321–e. doi: 10.2196/10321 PMC6329896 30632972

[37] Sun C Malcolm JC Wong B Shorr R Doyle M-A . Improving glycemic control in adults and children with type 1 diabetes with the use of smart-phone based mobile applications: A systematic review. Can. J. Diabetes (2018) 43(1): 51–8. doi: 10.1016/j.jcjd.2018.03.010 30026048

[38] Kim EK Kwak SH Jung HS Koo BK Moon MK Lim S . The effect of a smartphone-based, patient-centered diabetes care system in patients with type 2 diabetes: A randomized, controlled trial for 24 weeks. Diabetes Care (2019) 42(1):3–9. doi: 10.2337/dc17-2197 30377185

[39] Gunawardena KC Jackson R Robinett I Dhaniska L Jayamanne S Kalpani S . The influence of the smart glucose manager mobile application on diabetes management. J. Diabetes Sci. Technology. (2019) 13(1):75–81. doi: 10.1177/1932296818804522 PMC6313277 30264583

[40] Kerfoot BP Gagnon DR McMahon GT Orlander JD Kurgansky KE Conlin PR . A team-based online game improves blood glucose control in veterans with type 2 diabetes: A randomized controlled trial. Diabetes Care (2017) 40(9):1218–25. doi: 10.2337/dc17-0310 28790131

[41] Frias J Virdi N Raja P Kim Y Savage G Osterberg L . Effectiveness of digital medicines to improve clinical outcomes in patients with uncontrolled hypertension and type 2 diabetes: Prospective, open-label, cluster-randomized pilot clinical trial. J. Med. Internet Res. (2017) 19(7):e246–e. doi: 10.2196/jmir.7833 PMC5527253 28698169

[42] Kleinman NJ Shah A Shah S Phatak S Viswanathan V . Improved medication adherence and frequency of blood glucose self-testing using an m-health platform versus usual care in a multisite randomized clinical trial among people with type 2 diabetes in India. Telemedicine J. e-Health: Off. J. Am. Telemedicine Assoc. (2017) 23(9):733–40. doi: 10.1089/tmj.2016.0265 28328396

[43] Quinn CC Shardell MD Terrin ML Barr EA Park D Shaikh F . Mobile diabetes intervention for glycemic control in 45- to 64-Year-Old persons with type 2 diabetes. J. Appl. Gerontology: Off. J. South. Gerontological Society. (2016) 35(2):227–43. doi: 10.1177/0733464814542611 25098253

[44] Crowley MJ Edelman D McAndrew AT Kistler S Danus S Webb JA . Practical telemedicine for veterans with persistently poor diabetes control: A randomized pilot trial. Telemedicine J. e-Health: Off. J. Am. Telemedicine Assoc. (2016) 22(5):376–84. doi: 10.1089/tmj.2015.0145 26540163

[45] Kardas P Lewandowski K Bromuri S . Type 2 diabetes patients benefit from the COMODITY12 mHealth system: Results of a randomised trial. J. Med. Systems. (2016) 40(12):259. doi: 10.1007/s10916-016-0619-x 27722974

[46] Fukuoka Y Gay CL Joiner KL Vittinghoff E . A novel diabetes prevention intervention using a mobile app: A randomized controlled trial with overweight adults at risk. Am. J. Prev. Med. (2015) 49(2):223–37. doi: 10.1016/j.amepre.2015.01.003 PMC4509889 26033349

[47] Block G Azar KM Romanelli RJ Block TJ Hopkins D Carpenter HA . Diabetes prevention and weight loss with a fully automated behavioral intervention by email, web, and mobile phone: A randomized controlled trial among persons with prediabetes. J. Med. Internet Res. (2015) 17(10):e240–e. doi: 10.2196/jmir.4897 PMC4642405 26499966

[48] Wayne N Perez DF Kaplan DM Ritvo P . Health coaching reduces HbA1c in type 2 diabetic patients from a lower-socioeconomic status community: A randomized controlled trial. J. Med. Internet Res. (2015) 17(10):e224. doi: 10.2196/jmir.4871 26441467 PMC4642794

[49] Shahid M Mahar SA Shaikh S Shaikh Z-u . Mobile phone intervention to improve diabetes care in rural areas of Pakistan: A randomized controlled trial. J. Coll. Physicians Surgeons - Pakistan: JCPSP. (2015) 25(3):166–71. 25772954

[50] Greenwood DA Blozis SA Young HM Nesbitt TS Quinn CC . Overcoming clinical inertia: A randomized clinical trial of a telehealth remote monitoring intervention using paired glucose testing in adults with type 2 diabetes. J. Med. Internet Res. (2015) 17(7):e178–e. doi: 10.2196/jmir.4112 PMC4527012 26199142

[51] Drion I Pameijer LR van Dijk PR Groenier KH Kleefstra N Bilo HJG . The effects of a mobile phone application on quality of life in patients with type 1 diabetes mellitus: A randomized controlled trial. J. Diabetes Sci. Technology. (2015) 9(5):1086–91. doi: 10.1177/1932296815585871 PMC4667348 25963412

[52] Kirwan M Vandelanotte C Fenning A Duncan MJ . Diabetes self-management smartphone application for adults with type 1 diabetes: Randomized controlled trial. J. Med. Internet Res. (2013) 15(11):e235–e. doi: 10.2196/jmir.2588 PMC3841374 24225149

[53] Scottish Intercollegiate Guidelines Network (SIGN) . Methodology checklist 2 - randomised controlled trials . Available at: https://www.sign.ac.uk/what-we-do/methodology/checklists/.

[54] Cui M Wu X Mao J Wang X Nie M . T2DM self-management *via* smartphone applications: A systematic review and meta-analysis. PloS One (2016) 11(11):e0166718–e. doi: 10.1371/journal.pone.0166718 PMC5115794 27861583

[55] Fu H McMahon SK Gross CR Adam TJ Wyman JF . Usability and clinical efficacy of diabetes mobile applications for adults with type 2 diabetes: A systematic review. Diabetes Res. Clin. practice. (2017) 131:70–81. 10.1016/j.diabres.2017.06.016 28692830

[56] Wu Y Yao X Vespasiani G Nicolucci A Dong Y Kwong J . Mobile app-based interventions to support diabetes self-management: A systematic review of randomized controlled trials to identify functions associated with glycemic efficacy. JMIR MHealth UHealth. (2017) 5(3):e35. doi: 10.2196/mhealth.6522 28292740 PMC5373677

[57] Mao Y Lin W Wen J . Impact and efficacy of mobile health intervention in the management of diabetes and hypertension: A systematic review and meta-analysis. BMJ Open Diabetes Res. Care (2020) 8:e001225. doi: 10.1136/bmjdrc-2020-001225 PMC7523197 32988849

[58] Verma D Bahurupi Y Kant R Singh M Aggarwal P Saxena V . Effect of mHealth interventions on glycemic control and HbA1c improvement among type II diabetes patients in Asian population: A systematic review and meta-analysis. Indian J. Endocrinol. Metab. (2021) 25(6):484–92. 10.4103/ijem.ijem_387_21 PMC8959192 35355920

[59] Meyerowitz-Katz G Ravi S Arnolda L Feng X Maberly G Astell-Burt T . Rates of attrition and dropout in app-based interventions for chronic disease: systematic review and meta-analysis. J. Med. Internet Res. (2020) 22(9):e20283. 32990635 10.2196/20283 PMC7556375

[60] Cramer H Haller H Dobos G Lauche R . A systematic review and meta-analysis estimating the expected dropout rates in randomized controlled trials on yoga interventions. Evid Based Complement Alternat Med. (2016) 2016:5859729. doi: 10.1155/2016/5859729 27413387 PMC4927989

[61] Rohden AI Benchaya MC Camargo RS Moreira TC Barros HMT Ferigolo M . Dropout prevalence and associated factors in randomized clinical trials of adolescents treated for depression: Systematic review and meta-analysis. Clin. Ther. (2017) 39(5):971–992.e4. doi: 10.1016/j.clinthera.2017.03.017 28476404

[62] Gersh E Hallford DJ Rice SM Kazantzis N Gersh H Gersh B . Systematic review and meta-analysis of dropout rates in individual psychotherapy for generalized anxiety disorder. J. Anxiety Disord. (2017) 52:25–33. doi: 10.1016/j.janxdis.2017.10.001 29028610

[63] Pfammatter A Mitsos A Wang S Hood S Spring B . Evaluating and improving recruitment and retention in an mHealth clinical trial: An example of iterating methods during a trial. MHealth (2017) 3:49. doi: 10.21037/mhealth.2017.09.02 29184901 PMC5682361

[64] Fleming T Bavin L Lucassen M Stasiak K Hopkins S Merry S . Beyond the trial: Systematic review of real-world uptake and engagement with digital self-help interventions for depression, low mood, or anxiety. J. Med. Internet Res. (2018) 20(6):e199. doi: 10.2196/jmir.9275 29875089 PMC6010835

[65] Elbert SP Dijkstra A Oenema A . A mobile phone app intervention targeting fruit and vegetable consumption: The efficacy of textual and auditory tailored health information tested in a randomized controlled trial. J. Med. Internet Res. (2016) 18(6):e147. doi: 10.2196/jmir.5056 27287823 PMC4920964

[66] Lee K Kwon H Lee B Lee G Lee JH Park YR . Effect of self-monitoring on long-term patient engagement with mobile health applications. PloS One (2018) 13(7):e0201166. doi: 10.1371/journal.pone.0201166 30048546 PMC6062090

[67] Mak WW Tong AC Yip SY Lui WW Chio FH Chan AT . Efficacy and moderation of mobile app-based programs for mindfulness-based training, self-compassion training, and cognitive behavioral psychoeducation on mental health: Randomized controlled noninferiority trial. JMIR Ment. Health (2018) 5(4):e60. doi: 10.2196/mental.8597 30309837 PMC6231823

[68] Guertler D Vandelanotte C Kirwan M Duncan MJ . Engagement and nonusage attrition with a free physical activity promotion program: The case of 10,000 steps Australia. J. Med. Internet Res. (2015) 17(7):e176. doi: 10.2196/jmir.4339 26180040 PMC4526999

[69] Wei W Zhao S Fu SL Yi L Mao H Tan Q . The association of hypoglycemia assessed by continuous glucose monitoring with cardiovascular outcomes and mortality in patients with type 2 diabetes. Front. endocrinology. (2019) 10:536. 10.3389/fendo.2019.00536 PMC6691179 31447782

[70] NICE . Evidence standards framework for digital health technologies. UK: NICE (2021). 10.1177/20552076211018617 PMC8236783 34249371

[71] Reeves BC Deeks JJ Higgins JP . Including non-randomized studies. Cochrane Handb. Syst. Rev. Interv. (2008) 1:391.

